# Nanoemulsion Improves the Anti-Inflammatory Effect of Intraperitoneal and Oral Administration of Carvacryl Acetate

**DOI:** 10.3390/ph17010017

**Published:** 2023-12-21

**Authors:** Rafael Limongi de Souza, Luíza Carolina França Opretzka, Mayara Castro de Morais, Camila de Oliveira Melo, Brunna Emanuelly Guedes de Oliveira, Damião Pergentino de Sousa, Cristiane Flora Villarreal, Elquio Eleamen Oliveira

**Affiliations:** 1 Laboratory of Synthesis and Drug Delivery, State University of Paraíba, Rua Horácio Trajano, SN, João Pessoa 58071-160, PB, Brazil; 2 Laboratório de Farmacologia e Terapêutica Experimental, Faculdade de Farmácia, Universidade Federal da Bahia, Rua Barão de Jeremoabo, 147, Ondina, Salvador 40170-115, BA, Brazilcfv@ufba.br (C.F.V.); 3 Department of Pharmaceutical Sciences, Federal University of Paraíba, João Pessoa 58051-970, PB, Brazildamiao_desousa@yahoo.com.br (D.P.d.S.)

**Keywords:** anti-inflammatory effect, carvacrol derivative, carvacryl acetate, essential oil, oral administration, nanoemulsion

## Abstract

Carvacryl acetate (CA) is a monoterpene obtained from carvacrol, which exhibits anti-inflammatory activity. However, its low solubility in aqueous media limits its application and bioavailability. Herein, we aimed to develop a carvacryl acetate nanoemulsion (CANE) and assess its anti-inflammatory potential in preclinical trials. The optimized nanoemulsion was produced by ultrasound, and stability parameters were characterized for 90 days using dynamic light scattering after hydrophilic–lipophilic balance (HLB) assessment. To evaluate anti-inflammatory activity, a complete Freund’s adjuvant-induced inflammation model was established. Paw edema was measured, and local interleukin (IL)-1β levels were quantified using ELISA. Toxicity was assessed based on behavioral changes and biochemical assays. The optimized nanoemulsion contained 3% CA, 9% surfactants (HLB 9), and 88% water and exhibited good stability over 90 days, with no signs of toxicity. The release study revealed that CANE followed zero-order kinetics. Dose–response curves for CA were generated for intraperitoneal and oral administration, demonstrating anti-inflammatory effects by both routes; however, efficacy was lower when administered orally. Furthermore, CANE showed improved anti-inflammatory activity when compared with free oil, particularly when administered orally. Moreover, daily treatment with CANE did not induce behavioral or biochemical alterations. Overall, these findings indicate that nanoemulsification can enhance the anti-inflammatory properties of CA by oral administration.

## 1. Introduction

It is well-established that chronic inflammatory diseases are one of the most significant causes of mortality worldwide, and a considerable proportion of adult deaths are related to diseases exhibiting inflammatory patterns, such as heart disease, stroke, cancer, diabetes mellitus, chronic kidney disease, and neurodegenerative conditions, according to the latest Global Health Metrics (GBD 2020) [1]. Pharmacological therapies for inflammatory diseases include nonsteroidal and steroidal anti-inflammatory drugs. However, in addition to controlling inflammation, chronic use of these drugs can cause foregut symptoms, peptic ulcers, small bowel enteropathy, and metabolic and endocrine disorders [2,3,4,5,6].

Current research endeavors are focused on developing new drugs with improved safety profiles and novel solutions. Medicinal chemistry has been widely applied as a strategy for drug development, including the use of semisynthesis of natural sources [7,8,9]. Carvacrol is a phenolic monoterpene found in the aerial parts of plants of the genus *Origanum*, with anti-inflammatory properties [10,11,12]. However, it has relatively high toxicity compared to other esters and phenols [13]. Carvacryl acetate (CA), a semisynthetic monoterpenic ester derived from carvacrol with properties similar to its precursors and less toxicity, exhibits anti-inflammatory activity by reducing cytokine levels, neutrophil migration, and anti-nociceptive activity [13,14]. The oral lethal dose (LT_50_) of CA exceeds 2000 mg/kg in mice, thus exhibiting preclinical safety when compared with its precursor carvacrol and other monoterpenes [15,16]. The safety and efficacy of CA, compared with the significant side effects and low efficacy of the anti-inflammatory drugs currently available on the market, make it an excellent candidate with which to develop a new anti-inflammatory medicine. Despite its observed therapeutic activity, CA is a lipophilic molecule, which affects its bioavailability and pharmacological effect in vivo. Nanoemulsification can be a strategy to overcome this limitation and increase the bioavailability of CA by oral administration.

Nanoemulsion is a type of system commonly used to increase the solubility, bioavailability, and sustained release of essential oils. Nanoemulsions are colloidal dispersions of two immiscible liquids stabilized using surfactants [17,18,19,20,21]. Nanoemulsions are often used to increase chemical and physical stability, minimize organoleptic changes, and improve the biological activity of essential oils [10,22,23]. This system exhibits small droplet sizes, improving the solubility of hydrophobic drugs such as CA. Orally administered nanoemulsions can afford an increased drug dissolution rate, along with enhanced systemic solubility and bioavailability [24]. Another advantage of nanoemulsions is their drug release profile and absorption in the gastrointestinal tract [25].

Nanoemulsions have been developed and employed to assess the improved anti-inflammatory activity of other oils, including the precursor of CA, carvacrol [10,21,26]. However, nanosystems containing CA are yet to be developed to establish in vivo anti-inflammatory activity.

Previously, our group demonstrated that the nanoemulsification process improves the anthelmintic activity of CA via oral administration using a model of mice infected with *Schistosoma mansoni* [27]. In the present work, the influence of the mixture of surfactants on the stability of the CA nanoemulsion (CANE) was studied. The optimized nanoemulsion was used to evaluate its potential to improve the pharmacological properties of CA in an inflammation model. Further, toxicological studies were carried out in mice after oral administration of CA and CANE to assess their toxicological profile.

## 2. Results and Discussion

### 2.1. Spectroscopic Data of CA

The CA appeared as a yellow oil with 76% yield, TLC (9:1 hexane/EtOAc), Rf = 0.75; ^1^H-NMR (500 MHz, CDCl3) δH 7.17 (d, *J* = 10.0 Hz, 1H, H-6), 7.04 (dd, *J* = 10.0 Hz, 2.0 Hz, 1H, H-3), 6.89 (d, *J* = 2.0, 1H, H-4), 2.91 (hept, *J* = 8.5 Hz, 1H, H-7), 2.33 (s, 3H, H-10), 2.16 (s, 3H, H-9), 1.26 (d, *J* = 8.5 Hz, 6H, H-8). ^13^C NMR (125 MHz, CDCl3, δ ppm): 169.4 (C-10), 149.4 (C-1), 148.1 (C-5), 131.0 (C-3), 127.2 (C-2), 124.2 (C-4), 119.8 (C-6), 33.6 (C-7), 24.0 (C-8), 20.9 (C-11), 15.9 (C-9). 

In the ^1^H NMR spectra, it was observed that hydrogen atoms **3** and **4** in the aromatic ring exhibited coupling constants of 8.0 Hz, indicating coupling between *ortho* hydrogens. Hydrogen atom **6** showed a doublet with coupling constant *(J)* of 1.6 Hz, suggesting coupling with H-**4.** While the H-**7** showed a *septet* (*J* = 6.8 Hz), indicating coupling with H-**8** (dublet, *J* = 6.8 Hz). Hydrogens **9** and **11** were observed as singlets. In the ^13^C NMR spectra, the chemical shifts revealed a signal close to δ C 169.38 ppm assigned to the carbonyl carbon (C-**10**), a signal around δ C 149.37 ppm assigned to aromatic carbon **1**, and a signal at δ C 148.15 ppm attributed to aromatic carbon **5**. Additionally, signals at approximately δ C 130.92 ppm, δ C 127.24 ppm, 124.28 ppm, and δ C 119.84 ppm were assigned to carbons **3**, **2**, **4**, and **6** of the aromatic ring, respectively. The other signals were from the more protected methyl and methine carbons (See Supplementary Material). These data confirm that the compound obtained was CA [28].

### 2.2. Effect of HLB Values

In the present study, we developed a nanoemulsion containing CA to circumvent the limitations of lipophilic compounds. The selection and proportion of surfactants are critical steps in developing nanoemulsions. Selected surfactants must afford an HLB approximating that of the oil fraction. 

The micro-emultocrit technique, a short-term stability analysis, is a simple and rapid strategy to determine the HLB of essential oils and establish ideal proportions of surfactants. This technique shows a good correlation with long-term stability assays using a very small sample volume [29,30]. The methodology consists of producing a batch of emulsions with varying surfactant proportions and applying a high gravitational force to the systems, where the HLB value of the most stable emulsion—the small creaming index (CI)—is considered the required HLB of the oil. 

**Figure 1:**
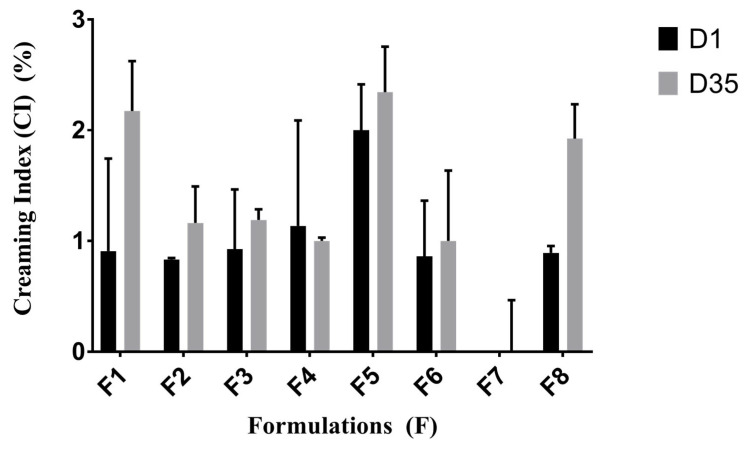
Analysis of creaming index (CI%) for the formulations containing carvacryl acetate (CA): short-term stability study performed using the micro-emultocrit technique on days (D) 1 (black column) and 35 (gray column).

High-HLB surfactants form more stable oil-in-water nanoemulsions than low-HLB surfactants. Water-in-oil nanoemulsions of natural oils have critical HLB values between 8 and 15 [31]. In this study, we formulated eight nanoemulsions by sonication using a mixture of surfactants containing Tween 80 and Span 80 presenting HLB values ranging between 8 and 15. This rational limitation in the HLB range studied saved costs and time as it reduced the number of experiments [32]. Tween 80 and Span 80 were chosen due their safety and biocompatibility and are described in the literature as suitable for preparing nanoemulsions via the sonication method [33]. Short-term stability results are shown in Figure 1.

The CI was used to evaluate the nanoemulsion stability. This index reflects the stability of the system, indirectly demonstrating the destabilization of droplets owing to creaming on the surface. The CI values are listed in Table 1. The lowest CI values were obtained on D1 and D35 between formulations and were related to formulation 7 (F7), corresponding to the surfactant mixture that afforded the HLB value of 9 required for CA.

### 2.3. Preparation and Characterization of CA Oil-Loaded Nanoemulsion

Based on the critical HLB study, we obtained a nanoemulsion containing a mixture of surfactants, with an HLB value approximating that required by CA. After that, 13 formulations were prepared, varying the amounts of surfactants (HLB 9) (3–18%) and CA (3–18%). The formulation that presented the smallest values of mean size and PDI and did not show phase separation after 7 days with the minimum quantity of surfactants was selected. The optimized nanoemulsion (CANE) consisted of 3% CA, 9% surfactants, and 88% water.

Nanoemulsions are thermodynamically unstable systems that can undergo structural changes during storage, such as creaming, coalescence, flocculation, Ostwald ripening, or phase separation [22,34]. To assess the stability of the formulated nanoemulsion, CANEs were evaluated over a 90-day period, and parameters such as droplet size, PDI, zeta potential, and pH were assessed (Table 2).

CANE exhibited an average droplet size of 101.50 ± 0.75 nm 90 days after preparation, with small variations on day 1. The time and potency used in high energy methods influence the nanoemulsion droplet sizes [35]. Ultrasonication was employed in the present study. This is a technique that applies disruptive forces to the system, causing turbulence, cavitation, and the breaking of larger oil droplets into smaller droplets [36]. The distribution of energy adequate to break the droplets on a nanometer scale is essential to produce a formulation with small size. This increases the surface area for surfactant adsorption, preventing the reaggregation of oil droplets. Preserving the droplet size over time is associated with good system stability. Some studies presenting stable nanoemulsions have employed Ostwald ripening inhibitors to avoid the gradual increase in droplets or other destabilization processes. In the present study, unlike other essential oil-containing nanoemulsions, CANE did not employ Oswald ripening inhibitors, such as medium-chain triglycerides, in the oil phase [10,37,38]. The proportion of surfactants with a critical HLB by CV allowed the development of a stable nanoemulsion, with 100% of the oil phase corresponding to the bioactive component. In some reports assessing carvacrol (CA precursor), stable nanoemulsions were produced without using Ostwald ripening inhibitors in the oil phase, based on surfactants with the critical HLB [23,39]. 

In addition, we evaluated PDI as another stability parameter. CANE showed PDI values ≤ 0.3 over 90 days. Notably, PDI shows the degree of uniformity of droplet size distribution within the system [40]. A gradual increase in the PDI value may indicate that the nanoemulsion has undergone destabilization, and the droplets in the system then aggregate to form larger droplets. This phenomenon was not observed in the present stability study. CANE had a PDI of 0.28 ± 0.01 at D90. Nanoemulsions with PDI values ≤ 0.3 and droplet sizes below 200 nm can be considered monodisperse systems [24]. The PDI value is associated with the method of preparation, the type of surfactant used, and the viscosity of the system. Tween 80 was previously described as a good stabilizer for carvacrol and other natural oil nanoemulsions [18,31]. 

Droplet size preservation and low variation in PDI may have been influenced by the adsorption kinetics of the surfactant mixture during formulation [41]. The use of surfactant mixtures can promote a synergistic effect in the interfacial region between the oil and water, improving the stability of nanoemulsions compared to the use of a single surfactant. The difference in headgroup size between Tween 80 and Span 80 can promote this synergistic effect between them, thus enhancing the stability of CANE [31,32]. 

**Figure 2:**
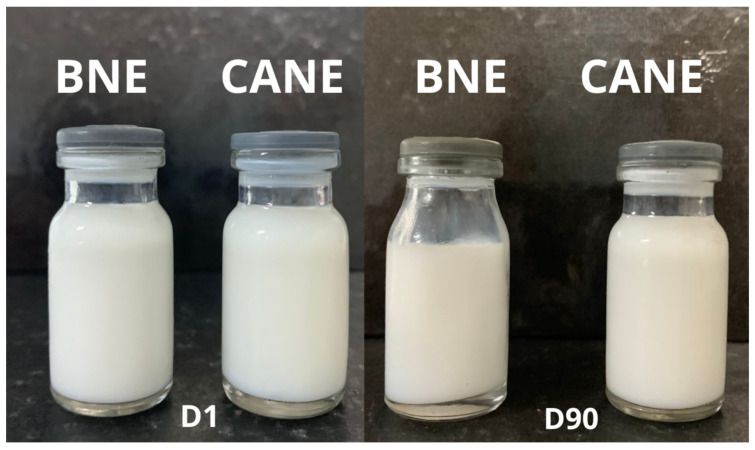
Macroscopic aspect of blank nanoemulsion (BNE) and carvacryl acetate nanoemulsion (CANE) on day 1 (D1) and day 90 (D90).

CANE presented a zeta potential of −28 mV at D1. After the first day of analysis, the values of zeta potential were close to −50 mV (Table 2). Zeta potential can serve as a partial indicator of system stability. It should be noted that markedly positive or negative values (above +30 mV or below −30 mV) may be preferable for promoting system stability, given the repulsion between droplets [42]. This increase in the negativity of zeta potential between the first analysis and others can be explained by the method of production used in this work. The sonication process can affect the components of the nanoemulsion, inducing the release of ions into the system. This release of ions can be present in the interfacial layer, inducing changes in the zeta potential of the system and justifying the differences found between the different days of analysis. This behavior can be observed in other studies with nanoemulsions, in which after the first moment of analysis the zeta potential values varied, differing from the values of the first day [43,44,45]. Moreover, this change does not reflect the stability of the CANE, considering that there was no substantial increase in droplet size, PDI variation, or macroscopic changes (Figure 2). 

The negative zeta potential values and small variation in pH values (close to 4) confirmed the superior stability of the nanoemulsion. In addition to its good physicochemical stability, CANE also preserved the oil content close to the initial concentrations, and showed an encapsulation efficiency of 97.77%, corresponding to a drug content of 29.3 mg/mL of CA. A nanosystem with a high encapsulation efficiency compared with other drug delivery systems used to encapsulate carvacrol or carvacryl acetate [23,46,47] was obtained in this study. This could be explained by the choice of the system, since nanoemulsions are efficient nanosystems with which to encapsulate hydrophobic molecules, as well as by the optimization of the mixture of surfactants (with the critical HLB of CA). 

### 2.4. Anti-Inflammatory Activity

#### 2.4.1. Anti-Inflammatory Profile of Intraperitoneally Administered CANE 

**Figure 3:**
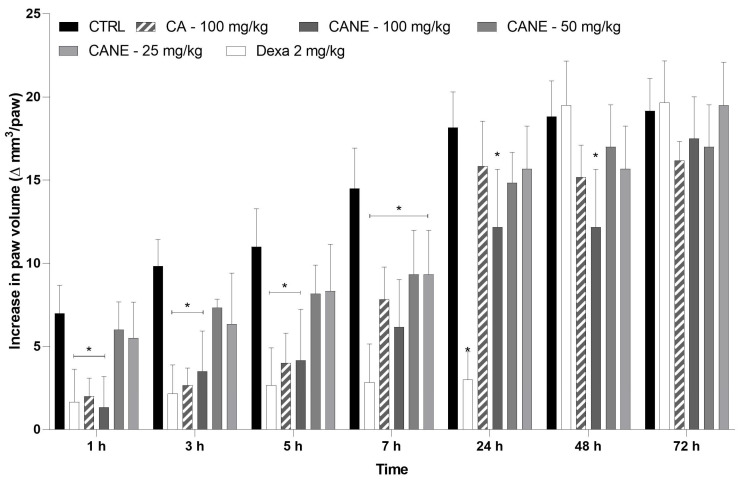
Effect of intraperitoneal carvacryl acetate nanoemulsion on CFA-induced paw edema. Different doses of carvacryl acetate nanoemulsion (CANE: 25, 50, and 100 mg/kg), free carvacryl acetate (CA: 100 mg/kg), blank nanoemulsion (CTRL, control group), or dexamethasone (Dexa; 2 mg/kg, reference drug) were administered intraperitoneally 40 min prior to CFA (time zero). The abscissa axis represents the time after CFA administration, while the ordinate axis represents the change in paw volume in mm^3^. Data are expressed the as mean ± standard deviation of six animals per group. * Statistical significance compared to the control group (*p* < 0.05), as determined by the two-way ANOVA test, followed by the Bonferroni post-test. CFA, complete Freund’s adjuvant.

In the present study, the anti-inflammatory effect of CANE was investigated in a mouse model of CFA-induced paw inflammation. CFA consists of heat-killed mycobacteria suspended in a mineral oil vehicle that produces chronic inflammation, often used to study the anti-inflammatory properties of novel compounds during drug discovery [48]. Following subcutaneous injection of CFA, mice developed gradually increasing paw edema that peaked after 24 h (Figure 3). 

**Figure 4:**
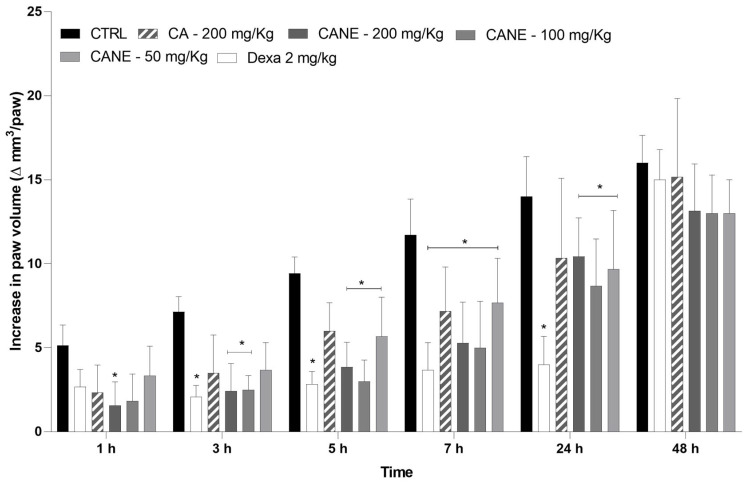
Effect of oral carvacryl acetate nanoemulsion on CFA-induced paw edema. Different doses of carvacryl acetate nanoemulsion (CANE; 50, 100, and 200 mg/kg), free carvacryl acetate (CA, 200 mg/kg), blank nanoemulsion (CTRL, control group), or dexamethasone (Dexa; 2 mg/kg, reference drug) were orally administered 1 h prior to the CFA injection (time zero). The abscissa axis represents the time after CFA administration, while the ordinate axis represents the change in paw volume in mm^3^. Data are expressed as the mean ± standard deviation of six animals per group. * Statistical significance compared to the control group (*p* < 0.05), as determined by the two-way ANOVA test, followed by the Bonferroni post-test. CFA, complete Freund’s adjuvant.

Intraperitoneal administration of free CA (100 mg/kg) induced an anti-edematogenic effect 1–7 h after CFA-induced paw inflammation (*p* < 0.05; Figure 4). Intraperitoneally administered CANEs (25, 50, and 100 mg/kg) induced a dose-dependent anti-edematogenic effect, lasting for up to 48 h after CFA administration (*p* < 0.05). The anti-inflammatory efficacy of CANE, as evidenced by the magnitude of the maximum effect, was similar to that afforded by free CA; however, the duration of this effect was prolonged, increasing from 7 h to 48 h. Importantly, CANE exhibited a longer-lasting anti-inflammatory effect than the gold standard drug dexamethasone (2 mg/kg). Nanoemulsions are colloidal systems that entrap and protect the lipophilic bioactive components of mixtures [25]. The preservation of CA droplets might favor the stability of the oil, increasing its bioavailability and enhancing its absorption across cells [49]. These factors may be associated with the ability of CANE to achieve superior anti-edematogenic activity gradually, compared with free oil and dexamethasone. The capacity of nanoemulsions to enhance the anti-inflammatory activity of lipophilic drugs via intraperitoneal injection has been previously reported [50,51,52].

#### 2.4.2. Anti-Inflammatory Profile of Orally Administered CANE 

The oral route is considered the most common and convenient route for drug administration. Oral routes have some advantages in terms of drug administration, affording patient adherence to administration [53]. Therefore, the effects of oral administration of CANE were evaluated and compared with the effect of CA and the gold standard, dexamethasone (Figure 4). 

Oral administration of CANE induced anti-edematogenic effects at all tested doses (50, 100, and 200 mg/kg; *p* < 0.05). At 200 mg/kg, CANE exhibited an anti-edematogenic effect starting 1 h after the CFA stimulus, which lasted for up to 24 h. In contrast, the anti-inflammatory effect mediated by free CA was significant (*p* < 0.05) only at 7 h, indicating that the nanoemulsion increased the time course of action of CA. Furthermore, oral administration of CA at doses ˂200 mg/kg did not induce anti-edematogenic effects, highlighting that the nanoemulsion improved the therapeutic dose range of CA when administered via the oral route. The efficacy of oral CANE was inferior to that of dexamethasone, although the duration of action was similar. Oral drug administration has several advantages; however, some challenges need to be overcome. Local factors, such as pH, mucus thickness, drug residence time, and microbiota, can influence the integrity and absorption of drugs in the gastrointestinal tract [53,54]. Stomach acidic conditions are the first and largest challenge encountered by oral drug formulations. Treatment with CANE (200 mg/kg) may prolong biological activity owing to the advantages afforded by nanoemulsion systems. First, the nanoemulsion preserves large amounts of CA along the gastrointestinal tract. Second, the formulated nanoemulsion increased the solubility of CA. Third, the nanoemulsion could increase the contact surface and thus enhance CA absorption. Finally, the prepared nanoemulsion facilitated modified CA release at the site of action. The ability of nanoemulsions to protect drugs against premature degradation, decrease hepatic first-pass metabolism, increase the interfacial area owing to droplet size, enhance the bioavailability of the molecule, and improve mucosal permeability has been previously established [55]. Moreover, previous studies have demonstrated the ability of nanoemulsions to prolong the anti-inflammatory activity of orally administered oils [21,56].

#### 2.4.3. Effect of CANE on the Local Production of the Pro-Inflammatory Cytokine IL-1β

**Figure 5:**
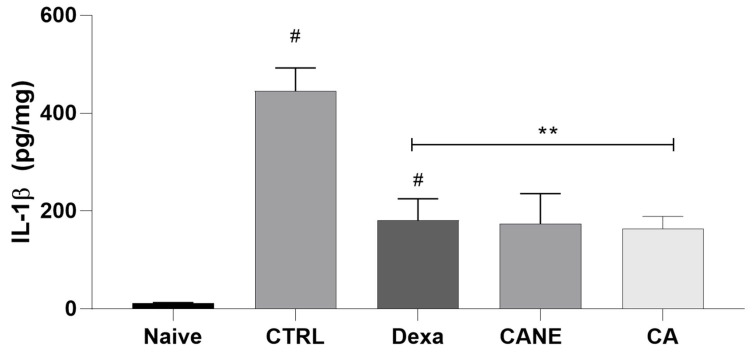
Effect of carvacryl acetate (CA) and carvacryl acetate nanoemulsion (CANE) on local production of interleukin (IL)-1β in the CFA-induced inflammation model. The ordinate axis represents IL-1β levels in mouse paw skin 7 h after intraplantar CFA administration. IL-1β levels, determined by ELISA, are expressed as picograms of cytokine per milligram of protein. CANE (200 mg/kg), CA (200 mg/kg), or blank nanoemulsion (CTRL, control group) were orally administered 1 h before CFA injection. Mice in the naïve group did not undergo experimental manipulation. Dexamethasone (Dexa; 2 mg/kg/intraperitoneal, 40 min before CFA) was administered in a maximum-effect protocol as the gold standard drug. Data are expressed as the mean ± standard deviation of six mice per group. # Statistical significance compared with the naïve group (*p* < 0.05); ** Statistical significance compared with the control group (*p* < 0.01), as determined by the 1-way ANOVA test, followed by the Tukey test. CFA, complete Freund’s adjuvant.

To corroborate the anti-inflammatory properties of CANE, we examined the modulatory effect of this nanoemulsion on the production of IL-1β, a primary pro-inflammatory cytokine, in the CFA model (Figure 5). Cytokine quantification was performed by harvesting the paw skin of mice from different experimental groups 7 h after CFA administration when both CANE and CA exerted anti-edematogenic effects. Oral treatment with CANE or CA at 200 mg/kg, 1 h before CFA injection, significantly reduced (*p* < 0.05) IL-1β levels in the inflamed paw, exhibiting efficacy similar to that of dexamethasone (2 mg/kg intraperitoneal) administered in a maximum-effect protocol. Consistent with the present data, CA has been shown to inhibit IL-1β and prostaglandin production [13,14]. IL-1β is a primary pro-inflammatory cytokine produced and released by different cell types, known to be involved in the development of signs and symptoms of inflammation [57]. After CFA administration, mice in the control group showed increased local levels of IL-1β, in line with previous reports, suggesting the key role of this cytokine in CFA-induced inflammation [58,59,60]. IL-1β can stimulate an inflammatory cascade, increasing cell recruitment, fever, vascular permeability, and prostaglandin production [61]. IL-1β interacts directly with the endothelium of blood vessels, increasing vascular permeability, which is involved in the genesis of edema. Furthermore, IL-1β induces the initiation of cyclooxygenase type 2 (COX-2) and inducible nitric oxide synthase (iNOS), leading to greater production of prostaglandin-E2 (PGE2) and nitric oxide (NO), which are also mediators of edema formation [62]. Considering that CANE and CA treatments reduced IL-1β levels in the inflamed paw, the inhibition of this cytokine may be responsible for the anti-edematogenic effect of these compounds. Indeed, the decrease in CFA-induced paw edema can be associated with carvacrol (CA precursor)-induced suppressed IL-1β production [61,63]. 

### 2.5. Systemic Toxicity

Repeated administration of anti-inflammatory drugs may lead to the development of serious side effects, such as gastrointestinal ulcers and renal disorders [64]. Furthermore, hepatotoxicity is one of the main causes of interruption in the drug development process or withdrawal after marketing [65]. Therefore, we next assessed the effects of daily CA and CANE administration on systemic metabolism by examining behavioral and serum biochemical parameters. Mice were orally administered CA (200 mg/kg), CANE (200 mg/kg), or vehicle for seven consecutive days. No clinically relevant changes in general behavior were observed during this period, with no deaths recorded. Body weight, as well as food and water intake, did not significantly differ between the groups. 

Urea and creatinine are commonly used as markers of renal function, playing an important role in assessing the risk and safety of new drugs and medications [66]. In CA- or CANE-treated mice, the values obtained for urea and creatinine serum levels did not differ from those of control or naïve mice. Moreover, liver function, assessed through liver enzyme (ALT and AST) levels, was not altered by treatments compared to controls (Table 3). Therefore, treatment with CA or CANE did not alter parameters indicative of liver or kidney toxicity, suggesting a favorable preclinical toxicological profile. These results indicate that daily oral treatment with CA or CANE did not induce systemic, renal, or hepatic toxicity, which was expected, given that CA has an LD_50_ ˃ 2000 mg/kg [15,16]. 

Mice were treated daily with a single oral dose of carvacryl acetate (CA; 200 mg/kg), carvacryl acetate nanoemulsion (CANE; 200 mg/kg), or blank nanoemulsion (BNE; control group) for 7 consecutive days. The naïve group underwent no experimental manipulation. Alanine transaminase (ALT), aspartate transaminase (AST), urea, and creatinine levels were determined. Data values are expressed as the mean ± standard deviation (SD) of six mice per group. ANOVA with Tukey’s post hoc test.

## 3. Materials and Methods

### 3.1. Materials

Sorbitan monostearate 80 (Span 80^®^), dexamethasone, and complete Freund’s adjuvant (CFA) were purchased from Sigma-Aldrich (St. Louis, MO, USA). Polysorbate 80 (Tween 80^®^) was provided by Vetec Química Fina Ltd. (Rio de Janeiro, Brazil). Mygliol 812^®^ was acquired from Sasol (Marl, Germany). The ELISA kit for interleukin (IL)-1β was acquired from R&D Systems (Minneapolis, MN, USA). Diagnostic kits for biochemical analyses were acquired from Labtest Diagnóstica (Lagoa Santa, Brazil). Sodium phosphate monobasic and dibasic (components of sodium phosphate buffer) were purchased from Sigma-Aldrich Co. (São Paulo, Brazil).

### 3.2. Synthesis and Characterization of CA

CA (98% purity) was obtained by acetylation of carvacrol as previously described [67]. Briefly, in a flask coupled to a condenser, carvacrol (5 g, 0.033 mol), pyridine (7.5 mL), and acetic anhydride (12.5 mL) were added. Then, the mixture was subjected to magnetic stirring and put under constant reflux for 24 h. The reaction mixture was poured into ice water (60 mL) and the product was extracted with chloroform (60 mL, three times) using a separatory funnel. The organic phase was treated with saturated copper sulfate (60 mL, three times), washed with water (60 mL, three times), and dried with anhydrous Na2SO4. After evaporation of the solvent under reduced pressure, the obtained product was subjected to column chromatography with silica gel using a mixture of hexane/ethyl acetate (95:5) as a mobile phase.

All procedures were followed by thin-layer chromatography (TLC). The visualization of the plates occurred through exposure to an ultraviolet irradiation lamp with a wavelength of 254 nm, through a MINERALIGHT device (model UVGL-58).

**Figure 6:**
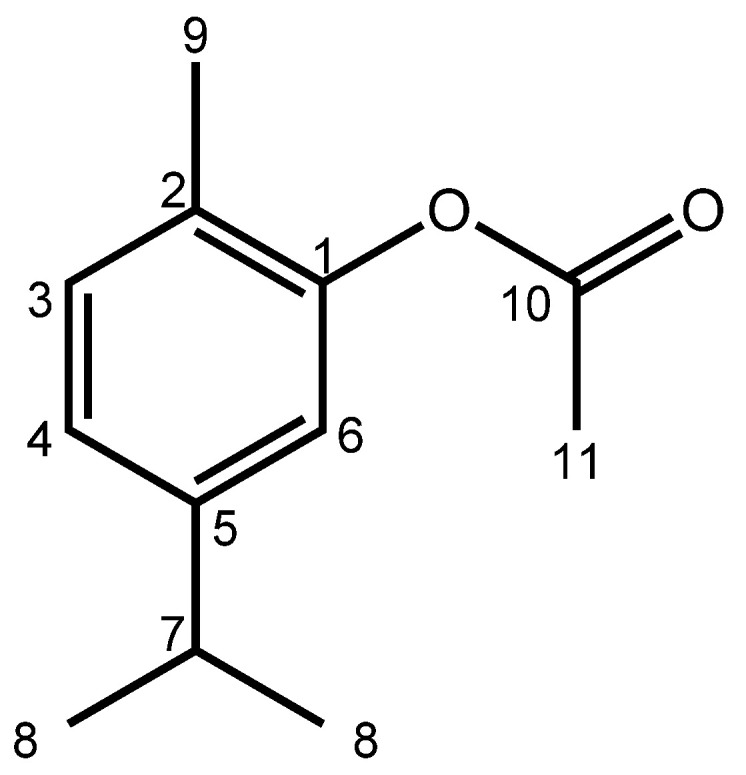
Chemical structure of carvacryl acetate.

The product (Figure 6) obtained was characterized by Hydrogen Nuclear Magnetic Resonance (^1^H NMR) and Thirteen Carbon Nuclear Magnetic Resonance (^13^C NMR) spectroscopy. The spectra were obtained using VARIAN-SYSTEM equipment operating at 500 MHz (^1^H) and 125 MHz (^13^C).

### 3.3. Hydrophilic–Lipophilic Balance (HLB) Assay

#### 3.3.1. Preparation of Nanoemulsions for HLB Analysis

To develop a stable nanoemulsion containing CA, it was necessary to discover the critical HLB value required by the oil. 

To develop tests for establishing the required HLB, we created a spreadsheet, including a mix of two surfactants: lipophilic (Span 80^®^, HLB = 4.3) and hydrophilic (Tween 80^®^, HLB = 15) (Supplementary Materials). Based on the proportion of surfactants used, 8 nanoemulsions were formulated, with HLB values ranging from 8 to 15.

After establishing the spreadsheet with surfactant proportions, nanoemulsions were prepared with different surfactant proportions, according to the spreadsheet, and subjected to four cycles of homogenization in a sonication apparatus (model QR200, Ultronique, São Paulo, Brazil) for 1 min at 300 W in an ice bath, subsequently followed by an ultrasonic bath for 1 min. The oil-in-water emulsion was composed of 5% (*w*/*w*) CA, 2% (*w*/*w*) mixed surfactants, and 93% (*w*/*w*) water.

#### 3.3.2. Micro-Emultocrit Technique

The short-term stability of prepared nanoemulsions with different HLB values was evaluated using the micro-emultocrit technique [30]. Heparin tubes were used, in which 75% of the volume of each tube was filled with nanoemulsions and placed in a microcentrifuge (Quimis, model Q10.500, São Paulo, Brazil) at 11,459× *g* for 10 min.

The test was performed at room temperature on day 1 (D1) and day 35 (D35) of storage. The creaming index (CI) of each formulation was evaluated after centrifugation. The formulation exhibiting the lowest CI was identified as the required HLB value for CA. All experiments were performed in triplicate.

#### 3.3.3. CI

The creaming rate was experimentally determined. Equation (1) was employed to measure this index. The CI values were obtained from the ratio of the total height of the cream layer (CC) to the total height of the emulsion layer (CT). The CC and CT were measured directly from a heparin-free capillary using a ruler.

(1) $$\%CI=\left(\frac{CC}{CT}\right)\times 100$$

### 3.4. CANE Formulation

The CANE was prepared using 3% (*w*/*w*) of CA, 9% (*w*/*w*) of mixed surfactants (Tween 80 and Span 80), and 88% (*w*/*w*) of water. The HLB value of the mixed surfactant used in this study was adjusted at nine. The mixture of components was emulsified by ultrasonication at 20 kHz (model QR200, Ultronique, São Paulo, Brazil), applying four cycles of sonication for 1 min each at 300 W, interspersed by an ultrasonic bath for 1 min. The sonicator was equipped with a titanium probe of 13 mm diameter. During the sonication, an ice bath was used to prevent overheating of the components. CA was replaced with Mygliol 812^®^ to prepare a blank nanoemulsion (BNE).

### 3.5. Stability Study

Stability assessments for CANE and BNE were performed for 90 days. The formulations were stored in 10 mL glass vials sealed with plastic caps and refrigerated at 4 ± 2 °C. Analyses were performed on days 1, 7, 15, 30, 45, 60, and 90 after formulation. On the day of analysis, droplet size, polydispersity index (PDI), zeta potential, pH, and macroscopic aspects were evaluated. The color and phase separation were examined in terms of macroscopic aspects.

### 3.6. Carvacryl Acetate Content

The CA content present in the nanoemulsion was observed after analysis using UV-Vis spectroscopy (Genesys 10S UV-Vis, Thermo Fischer Scientific, Dreieich, Germany) at 264 nm. Before analyzing the sample in the spectrometer, the formulation was diluted in methanol (1:200) and filtered using a 0.2 µm membrane filter (Kasvi, Brazil). The concentration of CA in the nanoemulsion was calculated using a calibration curve ((n = 9) y = 0.003044x + 0.01369; R² = 0.9972). All measurements were performed in triplicate. Drug loading content and drug recovery were calculated according to Equations (2) and (3), respectively.

(2) $$Drug\;loading\;content\left(\frac{mg}{mL}\right)=\frac{Amount\;of\;CA\;in\;the\;CANE}{Total\;amount\;of\;CANE}$$

(3) $$Drug\;recovery\;\%\left(\frac{w}{V}\right)=\left(\frac{Amount\;of\;CA\;in\;the\;CANE}{Amount\;of\;CA\;added}\right)\times 100$$

### 3.7. pH Measurement

The pH values of CANE and BNE were measured using a multipurpose autotitrator (model MPA-210, Tecnopon Instruments, Piracicaba, Brazil), previously calibrated with buffer solutions of pH 4.0 and 7.0.

### 3.8. Animals

Male Swiss mice were obtained from the Oswald Cruz Foundation (FIOCRUZ; Salvador, Bahia, Brazil) to establish the animal model. Animals (25–30 g) were housed in temperature-controlled rooms (22–25 °C) under a 12:12 h light–dark cycle, with access to water and food ad libitum until the initiation of experimentation. The experiments were approved by the Ethics Committee for Animal Experimentation of the Federal University of Bahia (CEUA/ICS 135/2018), and all behavioral tests were performed between 07:00 and 16:00. Groups of six animals were used for each experimental test.

### 3.9. Inflammatory Model

Mice were lightly anesthetized using halothane and administered a subcutaneous injection of CFA (20 μL, 1 mg/mL; time zero) in the right plantar region [63]. CFA consisted of 1 mg/mL heat-killed *Mycobacterium tuberculosis* in 85% paraffin oil and 15% mannidemonoleate. Forty minutes prior to the CFA injection, the animals were treated with CA (100 mg/kg), CANE (25, 50, and 100 mg/kg), or BNE (control group) via the intraperitoneal route. In another experimental set, animals were orally treated with CA (200 mg/kg), CANE (50, 100, and 200 mg/kg), or BNE, 1 h prior to the CFA injection. CA was dissolved in 5% dimethyl sulfoxide (DMSO) in sterile water, whereas CANE and BNE were dispersed in sterile water. Mice were administered dexamethasone, a gold standard drug, intraperitoneally (2 mg/kg) or orally (2 mg/kg), before CFA. Paw edema and local production of IL-1β were evaluated using a plethysmometer and ELISA, respectively, as described below.

### 3.10. Plethysmometer Test

CFA-induced edema was quantified by measuring the variation in paw volume using a plethysmometer device (Ugo, Basile, Comerio, Italy). The volume of each paw was measured prior to CFA administration (baseline) and 1, 3, 5, 7, 24, 48, and 72 h after CFA administration. Volume variation was determined by differences between measured paw volumes before and after CFA administration at pre-determined time points. 

### 3.11. Cytokine Measurement by ELISA

Paw cytokine levels were determined as previously described [63]. Mice were treated with CA (200 mg/kg), CANE (200 mg/kg), BNE, or dexamethasone (2 mg/kg; intraperitoneal) 1 h before intraplantar CFA injection. After 7 h, animals in each experimental group were deeply anesthetized and euthanized by cervical dislocation before collection of the paw skin. Next, the harvested sample was weighed, and for each 10 mg of tissue, 100 μL of cytokine extraction buffer (0.4 M NaCl, 0.05% tween 20, 0.5% bovine serum albumin, 0.1 mM PMSF, 0.1 mM benzethonium chloride, 10 mM EDTA, and 20 KI aprotinin diluted in 1× phosphate-buffered saline) was added. The harvested skin sample was immediately ground in a ball mill (TissueLyser II, Qiagen, Germantown, MD, USA) and centrifuged at 10,000× *g* for 10 min at 4 °C. The supernatant was collected, an aliquot was used to quantify total proteins (Bradford, 1976), and the remainder was stored in a freezer at −80 °C for subsequent cytokine quantification. Cytokine levels were quantified using mouse ELISA immunoassay kits for IL-1β. Results are expressed as picograms of cytokines per milligram of protein.

### 3.12. Toxicity Assessment

Signs of toxicity, mortality, weight, water, and feed consumption, general behavior, and blood biochemical parameters (creatinine, urea, alanine aminotransferase [AST], and aspartate aminotransferase [ALT] levels) were evaluated after daily treatment with CA or CANE. Animals received a daily oral dose of CA (200 mg/kg), CANE (200 mg/kg), or vehicle for seven consecutive days. Twenty-four hours after the completion of treatment, the animals were anesthetized by intraperitoneally administering a combination of 5% ketamine hydrochloride and 2% xylazine hydrochloride in equal parts. After induction of general anesthesia, a cardiac puncture was performed, and approximately 600 μL of total blood was collected. To establish the biochemical profile, 200 μL of blood without anticoagulant was centrifuged at 4750× *g* for 10 min at room temperature to obtain serum. Subsequently, serum was quickly collected and frozen at −70 °C. Biochemical analyses were performed using spectrophotometry, a Bioplus-200 semi-automatic biochemical analyzer, and standardized diagnostic kits (Labtest Diagnóstica) to determine the concentrations of ALT, AST, urea, and creatinine in collected samples.

### 3.13. Statistical Analysis

Data are presented as the mean ± standard deviation of six animals in each group. Comparisons between treatments were performed using one-way ANOVA, followed by Tukey’s test or, for repeated measures, two-way ANOVA followed by Bonferroni’s test, as deemed appropriate. All data were analyzed using GraphPad Prism version 8.0 (GraphPad, San Diego, CA, USA). Statistical significance was set at *p* < 0.05. 

## 4. Conclusions

In this study, we obtained a carvacryl acetate nanoemulsion with size close to 100 nm, which was stable for over 90 days, using a mixture of Tween 80 and Spam 80 with a HLB of 9. CA exhibited anti-inflammatory activity via both routes of administration, although oral efficacy was lower than that afforded by intraperitoneal administration. Moreover, CANE improved the anti-inflammatory activity of pure CA. In addition, CA and its nanoemulsion showed no detectable toxicity with oral daily treatment. Collectively, these results indicate that the nanoemulsification improves the pharmacological properties of CA, reducing the dose range required to afford an oral anti-inflammatory effect without detectable toxic effects. Thus, the data presented in this study will serve as a basis for future research using natural and semisynthetic oil nanoemulsions for oral delivery.

## Figures and Tables

**Table 1:** Analysis of creaming index (%) for the short-term stability study.

Formulation	HLB Value	Creaming Index (%)		Macroscopic Aspect
		D1	D35	
F1	15	0.90	2.17	M + CR
F2	14	0.83	1.56	M + CR
F3	13	0.92	1.21	M + CR
F4	12	1.13	1.02	M + CR
F5	11	2.00	1.78	M + CR
F6	10	0.86	2.00	M + CR
F7	9	0.00	0.00	M
F8	8	0.89	2.38	M + CR

F, formulation; D, day; HLB, hydrophilic–lipophilic balance; M, milky aspect; CR, creaming.

**Table 2:** Stability parameters: mean droplet diameter, zeta potential, polydispersity index, and pH of carvacryl acetate nanoemulsion (CANE) over 90 days.

Parameters of Stability/Day of Analysis	Mean Droplet Diameter (nm)	Polydispersity Index	Zeta Potential (mV)	pH
D1	93.39 ± 1.10	0.30 ± 0.03	−28.06 ± 0.96	4.17 ± 0.02
D7	98.88 ± 0.48	0.27 ± 0.01	−51.53 ± 1.67	4.16 ± 0.01
D15	99.83 ± 1.04	0.27 ± 0.01	−50.06 ± 1.55	4.19 ± 0.02
D30	98.99 ± 0.53	0.27 ± 0.03	−49.50 ± 2.35	4.01 ± 0.04
D60	105.90 ± 3.31	0.30 ± 0.04	−44.90 ± 3.06	4.00 ± 0.03
D90	101.50 ± 0.75	0.28 ± 0.01	−55.70 ± 1.28	4.05 ± 0.01

**Table 3:** Effects of daily oral treatment with carvacryl acetate or carvacryl acetate nanoemulsion on murine serum biochemical markers.

Parameters	Naïve	BNE	CA	CANE
ALT (UI/L)	71.66 ± 11.21	70.50 ± 11.41	69.66 ± 8.23	68.00 ± 7.43
AST (UI/L)	98.33 ± 10.52	99.00 ± 12.53	102.16 ± 8.42	101.50 ± 8.32
Urea (mg/dL)	52.33 ± 5.16	50.00 ± 3.22	50.33 ± 5.27	49.33 ± 4.55
Creatinine (mg/dL)	0.38 ± 0.07	0.38 ± 0.05	0.37 ± 0.03	0.37 ± 0.05

## Data Availability

The data presented in this study are available on request from the corresponding author.

## Supplementary Materials

The following supporting information can be downloaded at: https://www.mdpi.com/article/10.3390/ph17010017/s1, Figure S1. ^1^H NMR (400 MHz, CDCl_3_) spectrum of carvacryl acetate (**1**). Figure S2. The extension of the ^1^H NMR (400 MHz, CDCl_3_) spectrum of carvacryl acetate (**1**). Figure S3. The extension of the ^1^H NMR (400 MHz, CDCl_3_) spectrum of carvacryl acetate (**1**). Figure S4. ^13^C NMR (100 MHz, CDCl_3_) spectrum of carvacryl acetate (**1**). Table S1. Hydrophilic-Lipophilic Balance (HLB) Spreadsheet Design in accordance with individual surfactant percentage.
