# Supplementary material for: Nanoemulsion Improves the Anti-Inflammatory Effect of Intraperitoneal and Oral Administration of Carvacryl Acetate
Source: Pharmaceuticals (Basel). 2023 Dec 21;17(1):17. doi: 10.3390/ph17010017 (PMC10821396; doi:10.3390/ph17010017)
Supplement: Supplementary file 1 [file pharmaceuticals-17-00017-s001.zip › pharmaceuticals-2759205-supplementary.pdf]

SUPPLEMENTARY MATERIAL

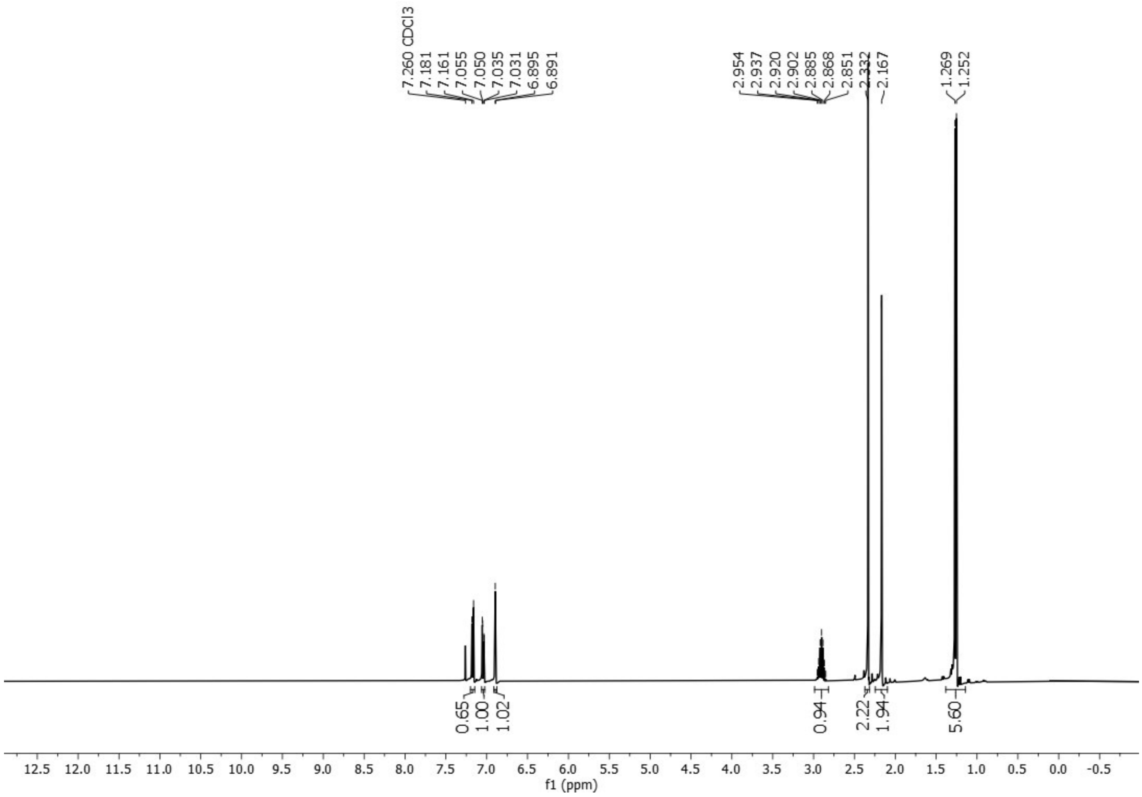

Figure S1. ^{1}H NMR (400 MHz, $CDCl_3$) spectrum of carvacryl acetate (**1**).

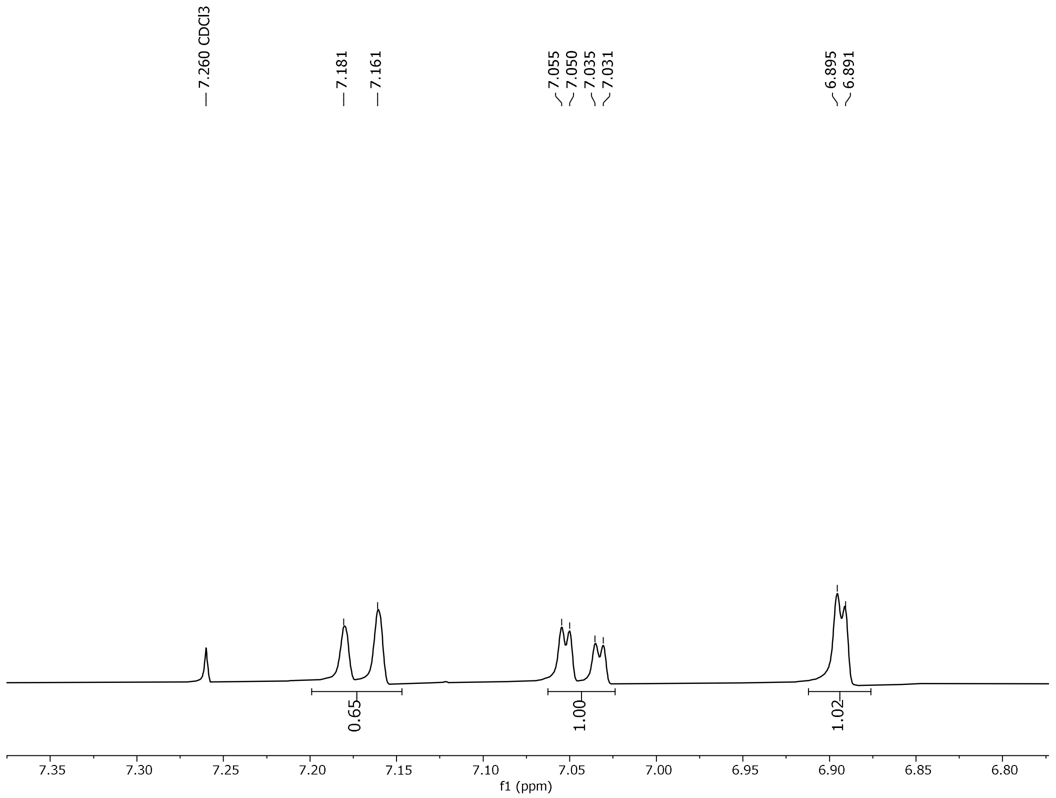

Figure S2. The extension of the ^{1}H NMR (400 MHz, $CDCl_3$) spectrum of carvacryl acetate (**1**).

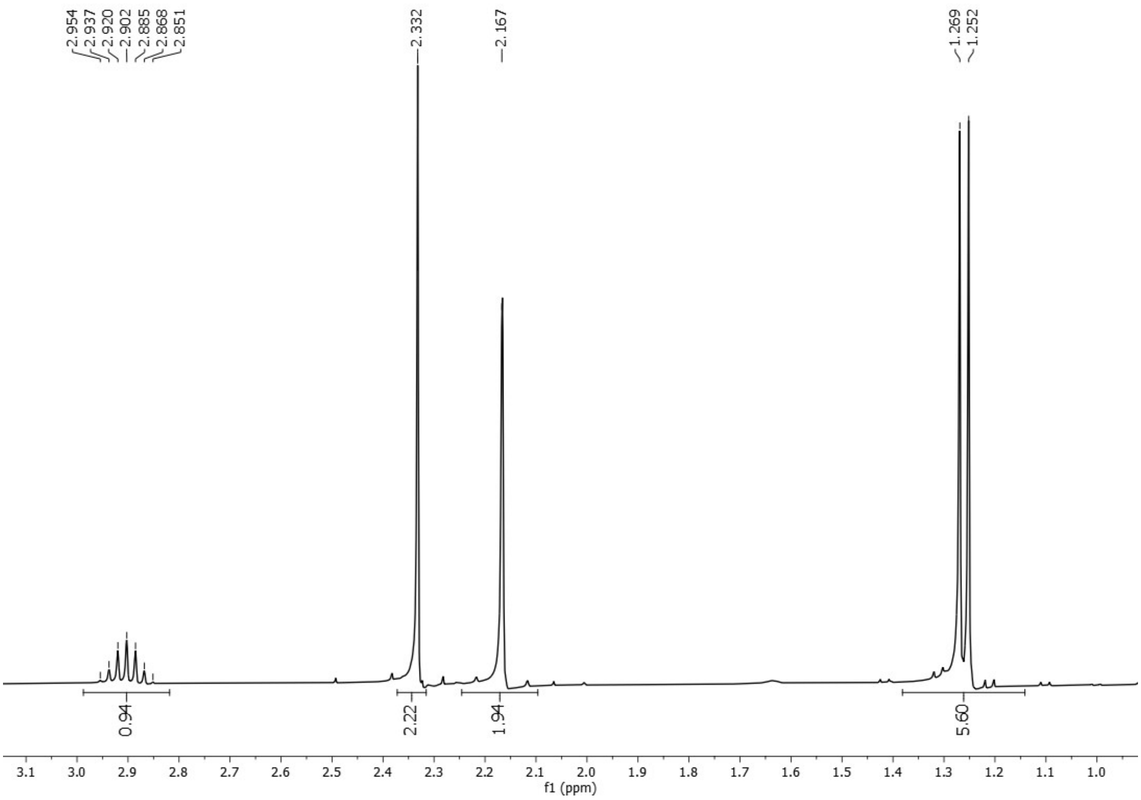

Figure S3. The extension of the ^{1}H NMR (400 MHz, $CDCl_3$) spectrum of carvacryl acetate (**1**).

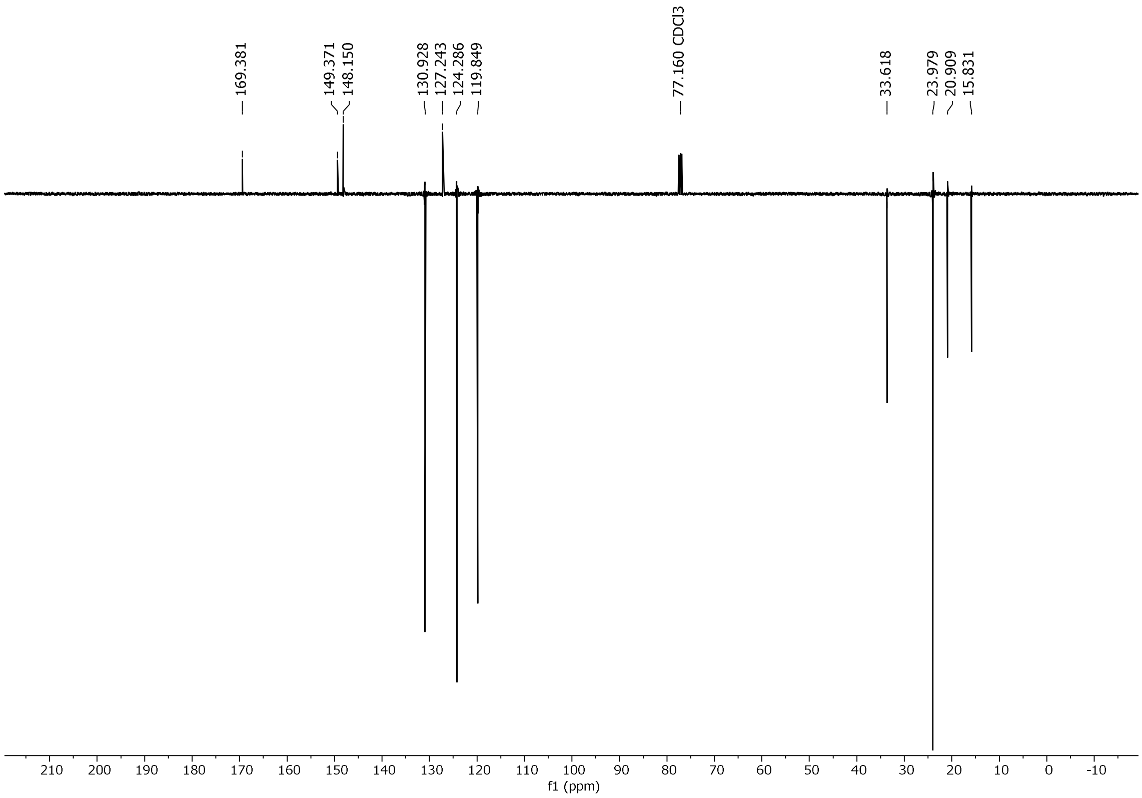

Figure S4. ^{13}C NMR (100 MHz, $CDCl_3$) spectrum of carvacryl acetate (**1**).

	TWEEN 80			SPAN 80			
**SAMPLE**	**(%W/W)**	**HLB CONTRIBUITION**	**W (g)**	**(%W/W)**	**HLB CONTRIBUITION**	**W (g)**	**FINAL HLB FORMULATION**
**F1**	100	15	**0,2**	0	0	**0**	15
**F2**	90,66	13,599	**0,18132**	9,34	0,40162	**0,01868**	14,00062
**F3**	81,31	12,1965	**0,16262**	18,69	0,80367	**0,03738**	13,00017
**F4**	71,97	10,7955	**0,14394**	28,03	1,20529	**0,05606**	12,00079
**F5**	62,62	9,393	**0,12524**	37,38	1,60734	**0,07476**	11,00034
**F6**	53,28	7,992	**0,10656**	46,72	2,00896	**0,09344**	10,00096
**F7**	43,93	6,5895	**0,08786**	56,07	2,41101	**0,11214**	9,00051
**F8**	34,58	5,187	**0,06916**	65,42	2,81306	**0,13084**	8,00006

Nanoemulsion Composition		
Mygliol	0,5	5%
Tween 80	0,2	1,20%
Span 80		0,80%
Água	9,3	93%
	g/mL	%

Table S1. Hydrophilic-Lipophilic Balance (HLB) spreadsheet design in accordance with individual surfactant percentages.
